# Meta-Analysis of SNP-Environment Interaction With Overlapping Data

**DOI:** 10.3389/fgene.2019.01400

**Published:** 2020-01-30

**Authors:** Qinqin Jin, Gang Shi

**Affiliations:** ^1^ State Key Laboratory of Integrated Services Networks, Xidian University, Xi'an, China; ^2^ Applied Science College, Taiyuan University of Science and Technology, Taiyuan, China

**Keywords:** meta-regression, meta-analysis, gene-environment interaction, overlapping data, correlation matrix

## Abstract

Meta-analysis, which combines the results of multiple studies, is an important analytical method in genome-wide association studies. In genome-wide association studies practice, studies employing meta-analysis may have overlapping data, which could yield false positive results. Recent studies have proposed models to handle the issue of overlapping data when testing the genetic main effect of single nucleotide polymorphism. However, there is still no meta-analysis method for testing gene-environment interaction when overlapping data exist. Inspired by the methods of testing the main effect of gene with overlapping data, we proposed an overlapping meta-regulation method to address the issue in testing the gene-environment interaction. We generalized the covariance matrices of the regular meta-regression model by employing Lin’s and Han’s correlation structures to incorporate the correlations introduced by the overlapping data. Based on our proposed models, we further provided statistical significance tests of the gene-environment interaction as well as joint effects of the gene main effect and the interaction. Through simulations, we examined type I errors and statistical powers of our proposed methods at different levels of data overlap among studies. We demonstrated that our method well controls the type I error and simultaneously achieves statistical power comparable with the method that removes overlapping samples *a priori* before the meta-analysis, i.e., the splitting method. On the other hand, ignoring overlapping data will inflate the type I error. Unlike the splitting method that requires individual-level genotype and phenotype data, our proposed method for testing gene-environment interaction handles the issue of overlapping data effectively and statistically efficiently at the meta-analysis level.

## Introduction

Numerous associations between human traits or diseases and single nucleotide polymorphisms (SNPs) have been identified by genome-wide association studies (GWAS) (Manolio, 2010). Meta-analysis combines the results from multiple studies to increase the effective sample size and statistical power of the association test (Fleiss, 1993; Borenstein et al., 2009). It has played an important role in finding the genetic architectures of complex traits and diseases.

Many meta-analysis methods are used in GWAS (Eleftheria and John, 2009). The fixed effect model is a commonly used method. It assumes that there are the same effect sizes across different studies. This method is effective if the heterogeneity among studies is small (Pfeiffer et al., 2009). Other methods, such as random effect models, are used in GWAS as well. They assume that the effect sizes of the studies follow a probability distribution due to the heterogeneity (Pereira et al., 2009). Recently, we proposed a new random effect method for testing the interaction between SNP and environment factor, which provides a higher power than the fixed effect methods when heterogeneity is large (Jin and Shi, 2019). The P-value based method (Fisher, 1967) was widely used earlier and has been abandoned because it does not include directions of effects under test; thus, it cannot provide an overall estimation of the effect size. The application of this method may lead to false positive results (Evangelou and Ioannidis, 2013). The Z scores method considers the direction of effect and its weight is estimated as the square root of the sample size of each study (Evangelou and Ioannidis, 2013). Bayesian methods (Kraft and Haiman, 2010) depend on the assumption of the prior distribution of the parameters and are usually computationally intensive. The subset method (Morris, 2011; Wen and Stephens, 2014) is similar to the fixed effect methods; however, it assumes that the effect exists only in a subset of the studies. All these classical methods assume that the studies have no overlapping samples, thus helping maintain independence among the summary statistics of the studies.

However, in GWAS practice, overlapping data between studies may occur. This may be caused inadvertently or intentionally by researchers. Spurious association may be achieved if overlapping data exist and are ignored in the meta-analysis (Lin and Sullivan, 2009; Han et al., 2016). Recently, meta-analysis methods, such as the P-value based method (Zaykin and Kozbur, 2010), subset method (Bhattacharjee et al., 2012), Bayesian method (Wen, 2014), fixed effect method (Lin and Sullivan, 2009), and random effect methods (Han and Eskin, 2011; Han et al., 2016) have been proposed for handling the overlapping data issue. All existing methods are for testing the SNP main effect. Lin’s method (Lin and Sullivan, 2009) is proposed for combining the results of case-control studies. It has been shown to yield higher and more robust power than the splitting method that removes the overlapped data in studies before calculating the study-level summary statistics. Han’s method (Han et al., 2016) involves modeling the covariance matrix of the estimated effects due to the overlapping data in fixed or random effect models and transforming the covariance matrix to be diagonal. The transformed matrix can then be synthesized by regular methods that assume independent data among studies.

Meta-regression (MR) (Xu et al., 2013) is an efficient meta-analysis method for testing SNP-environment interaction assuming independent data among studies. In MR, subjects in each study are divided into groups by the distribution of an environment variable. Then, the SNP main effects, standard errors, and the average environmental variables in each group are estimated using linear or logistic regressions. The SNP main effects and environmental variables across all groups are then collected and synthesized by MR. The overall main effect of the SNP, the effect of SNP-environment interaction, and the corresponding standard errors can be derived. The MR method is also shown to be robust when confounding effects exist (Shi and Nehorai, 2017).

Many complex diseases or traits are owing to the combination of effects of genetic factors, environment factors, and gene-environment interactions and involve in complex regulatory networks (Chen et al., 2019; Chen et al., 2019). Consider CDKN2A/B-rs10811661 as an example, which is associated with dyslipidemia. Researchers used CC/CT genotypes with a low-energy diet and a high frequency of exercise as the control group to study the effect of the interaction between rs10811661 gene polymorphism and energy intake and exercise on the level of blood lipid. The study found that the incidence of hypercholesterolemia was approximately 2 times higher in the TT genotype than in the control group and 1.5 times higher in the CC/CT genotype than in the control group (Mehramiz et al., 2018). The analysis of the genes and environment interactions can provide new insight into complex traits or disease mechanisms. However, a meta-analysis of SNP-environment interaction method with overlapping data does not exist currently. Data have to be split in studies such that every study contributes non-overlapped samples, i.e., the so-called splitting method. The splitting method requires the study-level genotype and phenotype data, which is usually unavailable for the meta-analysis. In addition, different ways of splitting samples may lead to different results.

In this paper, inspired by Lin’s method (Lin and Sullivan, 2009) and Han’s decoupling method (Han et al., 2016) for testing the SNP main effect, and based on MR, we propose the overlapping MR (OMR) method, which is a fixed effect MR model designed especially for handling overlapping data. The remainder of this paper is organized as follows: In the materials and methods section, we present the correlation matrices for the OMR method and then the method for testing the SNP-environment interaction. We also provide the relationship between MR and OMR. In the *Results* section, we simulate numerical examples and use them to examine the type I error and power of our method and the splitting method. We also show that the type I error is inflated with regular MR without considering overlapping samples. In the discussion and conclusion sections, we discuss the results and conclude the paper.

## Materials and Methods

Based on Lin’s and Han’s correlation structures (Lin and Sullivan, 2009; Han et al., 2016), we generalized regular MR model for independent studies to consider studies with correlated summary statistics due to overlapping data. To describe our method clearly, we first briefly introduce the regular MR method.

### Regular MR Method

Before the MR analysis, individuals in each study are first stratified into several groups according to their environmental measurements. The main effects of SNP at the group level can be estimated via linear regression as follows:

$$Y=\beta_{0}+\beta_{G}G+\beta_{E}E+\epsilon ,$$

where *Y* is a quantitative phenotype, *G* is the code of the SNP, and *E* is the environmental measurement.

Assume that $\hat{\beta }$ is the estimate of the SNP main effect, and ${\hat{\beta }}_{ij}\;$is the estimate of the SNP main effect for the *i*-th study and the *j*-th group where *i*= 1,2,…,*n*, *j*=1,2,…,*n_i_*, The symbol *n* is the number of studies and *n_i_* denotes the number of groups in the *i*-th study, and ${\hat{e}}_{ij}\;$denotes the standard error in the *j*-th group of the *i*-th study. The mean environmental measurement in the *j*-th group of the *i*-th study is *E_ij_*. *α* is the regression coefficient vector of interest. The symbol *X* is the interest matrix and *X_i_* is the interest matrix for the *i*-th study. *ε* is the standard error matrix and the *ε_i_* is the standard error matrix for *i*-th study. In MR, the SNP effect is regressed on the environmental factor as follows:

(1) $$\hat{\beta }=X\alpha +\epsilon ,$$

where

$$\begin{array}{c} \hat{\beta }=\left(\begin{array}{c} {\hat{\beta }}_{1}\\ \begin{array}{c} {\hat{\beta }}_{2}\\ \vdots \end{array}\\ {\hat{\beta }}_{n} \end{array}\right),\;{\hat{\beta }}_{i}=\left(\begin{array}{c} {\hat{\beta }}_{i1}\\ \begin{array}{c} {\hat{\beta }}_{i2}\\ \vdots \end{array}\\ {\hat{\beta }}_{in_{i}} \end{array}\right),X=\left(\begin{array}{c} X_{1}\\ \begin{array}{c} X_{2}\\ \vdots \end{array}\\ X_{n} \end{array}\right),X_{i}=\left(\begin{array}{cc} 1 & E_{i1}\\ \begin{array}{c} 1\\ \vdots \end{array} & \begin{array}{c} E_{i2}\\ \vdots \end{array}\\ 1 & E_{in_{i}} \end{array}\right),\\ \epsilon =\left(\begin{array}{c} \epsilon_{1}\\ \begin{array}{c} \epsilon_{2}\\ \vdots \end{array}\\ \epsilon_{n} \end{array}\right),\epsilon_{i}=\left(\begin{array}{c} \epsilon_{i1}\\ \begin{array}{c} \epsilon_{i2}\\ \vdots \end{array}\\ \epsilon_{in_{i}} \end{array}\right),\alpha =\left(\begin{array}{c} \alpha_{1}\\ \alpha_{2} \end{array}\right),\;\Sigma =\left(\begin{array}{ccc} \Sigma_{1} & \cdots & 0\\ \vdots & \ddots & \vdots \\ 0 & \cdots & \Sigma_{n} \end{array}\right),\\ \Sigma_{i}=\left(\begin{array}{ccc} {\hat{e}}_{i1} & \cdots & 0\\ \vdots & \ddots & \vdots \\ 0 & \cdots & {\hat{e}}_{in_{i}} \end{array}\right) \end{array}$$

and $\epsilon_{ij}\sim \text{N}\left(0,{\hat{e}}_{ij}\right),i=1,2,\ldots ,n,\;j=1,2,\ldots ,n_{i}$.

*α* and Cov(*α*) are estimated by (Xu et al., 2013; Shi and Nehorai, 2017).

(2) $$\begin{array}{c} \hat{\alpha }={\left(X^{\prime }\Sigma^{-1}X\right)}^{-1}X^{\prime }\Sigma^{-1}\hat{\beta }\\ {\hat{\alpha }}_{2}=\left(0,1\right)\;\hat{\alpha }\\ \text{Cov}\left(\hat{\alpha }\right)={\left(X^{\prime }\Sigma^{-1}X\right)}^{-1}\\ \text{Cov}{\left(\hat{\alpha }\right)}_{22}=\left(0,1\right){\left(X^{\prime }\Sigma^{-1}X\right)}^{-1}\left(\begin{array}{c} 0\\ 1 \end{array}\right) \end{array}$$

Under the null hypothesis H_0_:*α*_2_=0, Wald statistic for testing the SNP-environment interaction effect is ${\hat{\alpha }}_{2}/\text{Cov}{\left(\hat{\alpha }\right)}_{22}$, which follows a 1 degree of freedom (df) χ^2^ distribution. Under the null hypothesis of H_0_:*α*=0, the Wald statistic for testing joint effects of the SNP and the interaction is ${\hat{\alpha }}^{'}\text{Cov}{\left(\hat{\alpha }\right)}^{-1}\hat{\alpha }$, which follows a 2 df χ^2^ distribution.

The model (1) can be specified as any nonlinear function of the environmental variable as necessary. For example, to test quadratic SNP-environment interaction, the model can be formulated as

(3) $$\hat{\beta }=X^{N}\alpha^{N}+\epsilon^{N}$$

where

$$X^{N}=\left(\begin{array}{c} X_{1}^{N}\\ \begin{array}{c} X_{2}^{N}\\ \vdots \end{array}\\ X_{n}^{N} \end{array}\right),X_{i}^{N}=\left(\begin{array}{ccc} 1 & E_{i1} & E_{i1}^{2}\\ \begin{array}{c} 1\\ \vdots \end{array} & \begin{array}{c} E_{i2}\\ \vdots \end{array} & \begin{array}{c} E_{i2}^{2}\\ \vdots \end{array}\\ 1 & E_{in_{i}} & E_{in_{i}}^{2} \end{array}\right),\alpha^{N}=\left(\begin{array}{c} \alpha_{1}^{N}\\ \alpha_{2}^{N}\\ \alpha_{3}^{N} \end{array}\right).$$

The Wald statistic then follows a 2 df χ^2^ distribution when testing the two interaction effects simultaneously. The Wald statistic follows a 3 df χ^2^ distribution for testing the SNP main and interactions jointly (Xu et al., 2013).

### Overlapping MR Method

Inspired by the methods for testing the SNP main effect with overlapping data (Lin and Sullivan, 2009), based on regular MR, we propose the OMR model for testing the SNP-environment interaction when data among studies are overlapped.

We consider the kernel process for modeling the correlations due to the overlapping data. Following Lin’s recommendation, the covariance matrix under the correlated studies can be modeled as follows (Lin and Sullivan, 2009):

(4) $$\Omega =\Sigma^{1/2}C\Sigma^{1/2},$$

where *C* is the correlation matrix. The dimensions of this matrix *C* are related to the number of studies and the group number of each study. The details of the correlation matrix will be presented in the next section.

Alternatively, the variance covariance matrix can be generalized according to Han’s suggestion as follows (Han et al., 2016):

(5) $$\Omega =\text{diag}{\left(e^{\prime }{\left(\Sigma^{1/2}C\Sigma^{1/2}\right)}^{-1}\right)}^{-1}$$

where *e* is a vector of ones whose length is the sum of the number of groups among all studies. After this modification, the correlation matrix becomes a diagonal matrix. This matrix is highly likely to be positive semi-definite and the analysis of the positive semi-definite matrix is similar to the condition of case-control studies (Han et al., 2016).

Lin’s variance covariance matrix is equivalent to Han’s (Han et al., 2016). The variance covariance matrix based on Han’s formula (5) is more flexible. However, it is more computationally intensive. The method of Lin is simple in its mathematical form and calculation. In cases analyzing with existing programs that require studies to be independent, Han’s method can be applied.

### Correlation Matrices

Lin and Sullivan (2009) developed a correlation matrix *C* for incorporating correlations among summary statistics of studies due to the overlapping data. The correlation of studies *i* and *j* is given as follows:

(6) $$\gamma_{ij}\approx n_{ij}/\sqrt{n_{i}n_{j}},$$

where *n_i_* and *n_j_* are the numbers of studies *i* and *j*respectively, and *n_ij_* is the number of overlapped individuals between the *i*-th and *j*-th studies.

When considering the MR method, this correlation can be modeled as follows:

(7) $$\gamma_{i_{h}j_{k}}\approx n_{i_{h}j_{k}}/\sqrt{n_{i_{h}}n_{j_{k}}},$$

where $\;n_{i_{h}}$ and $n_{j_{k}}$ are the sample sizes of the *h*-th group of study *i* and the *k*-th group of study *j*, and $n_{i_{h}j_{k}}$ is the number of overlapping samples between them. In this correlation structure, the block matrix that corresponds to each study is an identity matrix; that is, the diagonal block matrices of the correlation matrix are all identity matrices.

### Hypothesis Testing

With the introduced correlation matrix, linear unbiased estimates $\hat{\alpha }$ and $\text{Cov}\left(\hat{\alpha }\right)$ can be found as follows (Becker and Wu, 2007):

(8) $$\begin{array}{c} \hat{\alpha }={\left(X^{\prime }\Omega^{-1}X\right)}^{-1}X^{\prime }\Omega^{-1}\hat{\beta }\\ {\hat{\alpha }}_{2}=\left(0,1\right)\hat{\alpha }\\ \text{Cov}\left(\hat{\alpha }\right)={\left(X^{\prime }\Omega^{-1}X\right)}^{-1}\\ \text{Cov}{\left(\hat{\alpha }\right)}_{22}=\left(0,1\right)\text{Cov}\left(\hat{\alpha }\right)\left(\begin{array}{c} 0\\ 1 \end{array}\right) \end{array}$$

Under the null hypothesis *α*_2_=0, the Wald statistic for testing the SNP-environment interaction effect is given as follows:

(9) $${\text{S}}_{\text{I}}={\text{α}}_{2}^{2}/\text{Cov}{\left(\hat{\alpha }\right)}_{22}$$

This statistic follows a 1 df χ^2^distribution.

Under null distribution *α*=0 the Wald statistics for testing the SNP and the interaction joint effects are given as follows:

(10) $${\text{S}}_{\text{J}}={\hat{\alpha }}^{2}/\text{Cov}\left(\hat{\alpha }\right)$$

which follows a 2 df χ^2^ distribution.

OMR method can also be extended to test nonlinear SNP-environment interaction for overlapping method. This process is similar with model (1), the Wald statistic for the test of SNP-environment interaction and quadratic SNP-environment interaction follows a 2 df χ^2^ distribution. The Wald statistic for testing the SNP, SNP-environment interaction, and quadratic SNP-environment interaction interactions jointly follows a 3 df χ^2^ distribution.

As can be seen, our models are generalized versions of the regular MR. When the data of studies are independent, correlation matrix *C* is an identity matrix, and the two covariance matrices become

(11) $$\Omega =\Sigma^{\frac{1}{2}}C\Sigma^{\frac{1}{2}}=\Sigma$$

and

(12) $$\Omega =\text{diag}{\left(e^{\prime }{\left(\Sigma^{1/2}C\Sigma^{1/2}\right)}^{-1}\right)}^{-1}=\Sigma$$

In this case, the covariance matrix is identical to that of the regular MR.

## Results

We evaluated the type I error to ensure that the false positive rate is appropriately controlled by our proposed OMR method when overlapping data exist, that is, whether the empirical type I error rate is close to the specified level. We compared our method with the splitting method and regular MR method, which did not consider overlapping data. The power was then compared at different levels of sample overlap. We considered two scenarios where there were 100 and 400 overlapping subjects between every two studies.

### Simulation

The quantitative phenotype *Y* was simulated as being related to *G* and *E,* which were the genotypes of the SNP and environment variables, respectively. The simulation model representing this relationship is given as follows:

$$Y=\beta_{G}G+\beta_{G\times E}G\times E+\beta_{E}E+\epsilon$$

Here, the SNP was assumed to have an additive genetic effect; the minor allele frequency was 0.3, and *G* was the code of SNP, which was the number of minor alleles. We generated random numbers by the runif function in R, then the values of *G* are determined by which intervals the random numbers fall into, and the intervals are determined by genotype frequency. Variable *E* was normally distributed, *E*~N(0,1). 10% of the variation in *Y* was explained by *β_E_E*. The fixed effects *β_G_* and *β*_*G*×*E*_ varied in our simulated datasets. The random error *ε* was normally distributed with zero mean and its variance was chosen such that phenotypic variance is unit. The environment variable and error term were generated by the rnorm function in R. In all our numerical experiments, we considered meta-analyses of data from 2, 3, 4, 5, and 6 studies, each of which had 1,000 unrelated individuals. In each study, we simulated three variables: the phenotype *Y*, environmental *E*, and genotype SNP. Across studies, there were 100 or 400 overlapping samples between any two studies. Under each simulation setup, data were generated with 1,000 replicates.

We divided 1,000 unrelated individuals in each study into five groups according to the distribution of *E*, before meta-analyses. In each group, we applied linear regression to estimate the main effects *β_G_*, its corresponding error *ε*, and the mean environment variable *E*. Meta-analysis were performed with 2, 3, 4, 5, and 6 studies.

#### Type I Error

**Figure 1:**
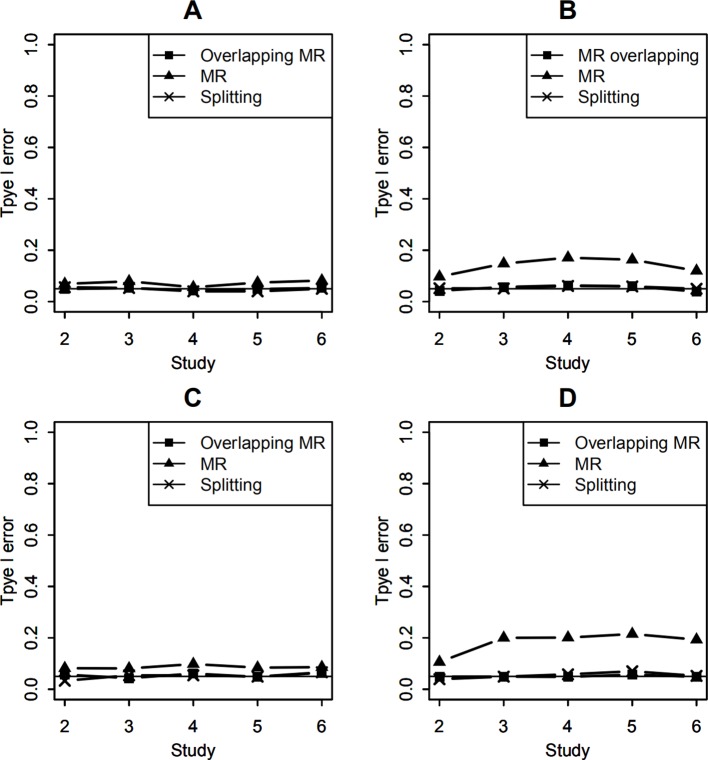
Type I error of testing SNP-environment interaction and jointly testing SNP main effect and the interaction. **(A, B)** are type I errors of the interaction test with 100 and 400 overlapping data, respectively. **(C, D)** are type I errors of the joint test with 100 and 400 overlapping data, respectively. Solid line with crosses is type I errors of the splitting method with 2, 3, 4, 5, and 6 studies. Solid line with filled squares is type I errors of OMR method with 2, 3, 4, 5, and 6 studies. Solid line with filled triangles is type I errors of the regular MR with 2, 3, 4, 5, and 6 studies when overlapping data is ignored.

To obtain the type I error of the interaction test, the effect of the SNP-environment interaction was set to be zero and the SNP main effect explained 0.5% variance of the trait variance. The empirical type I error of our method was calculated by transforming the covariance matrix with overlapping data into a diagonal matrix and then using regular MR. Under this simulation, the test of empirical type I error of our method followed a 1 df distribution. The empirical type I error of the splitting method with two studies was estimated by removing 100 or 400 overlapping subjects of study 1, and the data in study 2 were left unchanged. The empirical type I error of the splitting data method with 3, 4, 5, and 6 studies was estimated by discarding 100 or 400 overlapped subjects from each study. **Figures 1A, B** show the type I error rates of 2, 3, 4, 5, and 6 studies in the test of SNP-environment interaction with 100 and 400 overlapping subjects, respectively. We can see that both our method and the splitting data method yielded type I error results close to the specified 0.05 level. The regular MR method, which did not consider overlapping data, yielded inflated type I error rates. The greater the overlap, the more the inflation was.

To calculate the type I error rates of the joint test of the SNP main effect and the interaction, we set both the SNP and the SNP-environment interaction effects to be zeros. The Wald test statistics followed a 2 df χ^2^distribution. **Figures 1C, D** show the type I errors of the joint test under the null hypotheses. We can also see that the results of the two methods were around 0.05 as well; thus, both our OMR method and splitting method treated the overlapping data appropriately. The regular MR method in the joint test yielded a higher type I error than in the interaction test because it included more information on overlapping data.

**Figure 2:**
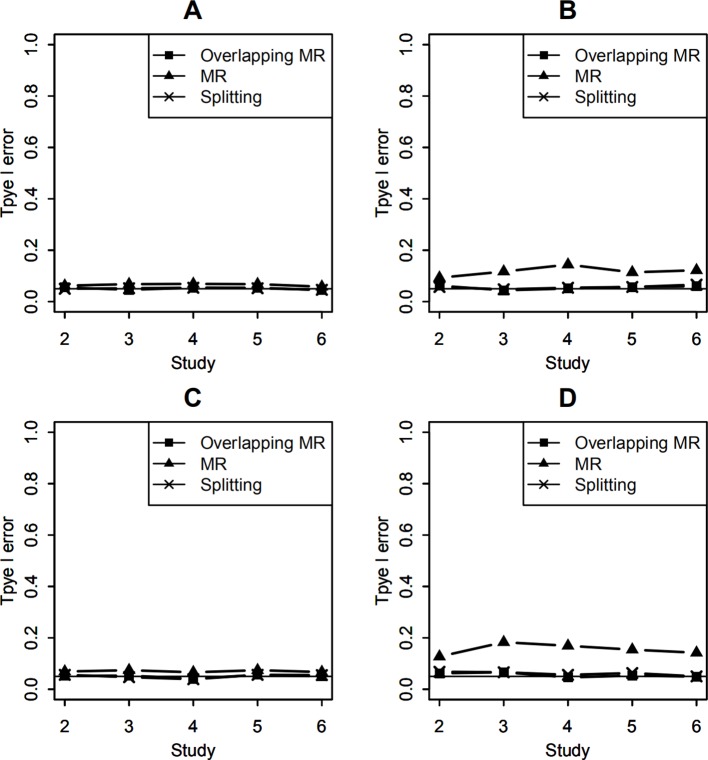
Type I error of testing SNP-environment interaction and jointly testing SNP main effect and the interaction with 6 studies of 1,000, 1,200, 1,400, 1,600, 1,800, 2,000 individuals, respectively. **(A, B)** are type I errors of the interaction test with100 and 400 overlapping data, respectively. **(C, D)** are type I errors of the joint test with 100 and 400 overlapping data, respectively. Solid line with crosses is type I errors of the splitting method with 2, 3, 4, 5, and 6 studies. Solid line with filled squares is type I errors of OMR method with 2, 3, 4, 5, and 6 studies. Solid line with filled triangles is type I errors of the regular MR with 2, 3, 4, 5, and 6 studies when overlapping data is ignored.

In real meta-analysis, sample sizes of studies vary and percentages of overlapping may be different for studies. Here, we set the sample sizes of the 6 studies as (1,000, 1,200, 1,400, 1,600, 1,800, 2,000). Let the effect of the SNP-environment interaction to be zero and the SNP main effect explained 0.5% of trait variance. Type I errors of testing the SNP-environment interaction are shown in **Figures 2A, B**, which represent results of testing the interaction with 100 and 400 overlapping individuals in each study, respectively. Setting both the SNP and the SNP-environment interaction effects to be zeros, we conducted joint tests for SNP and SNP-environment interaction. **Figures 2C, D** show type I errors of the joint test with 100 and 400 overlapping individuals, respectively. As the results in **Figure 1**, OMR and the splitting method control type I errors as expected, while inflated type I errors can be observed for the regular MR.

#### Power

**Figure 3:**
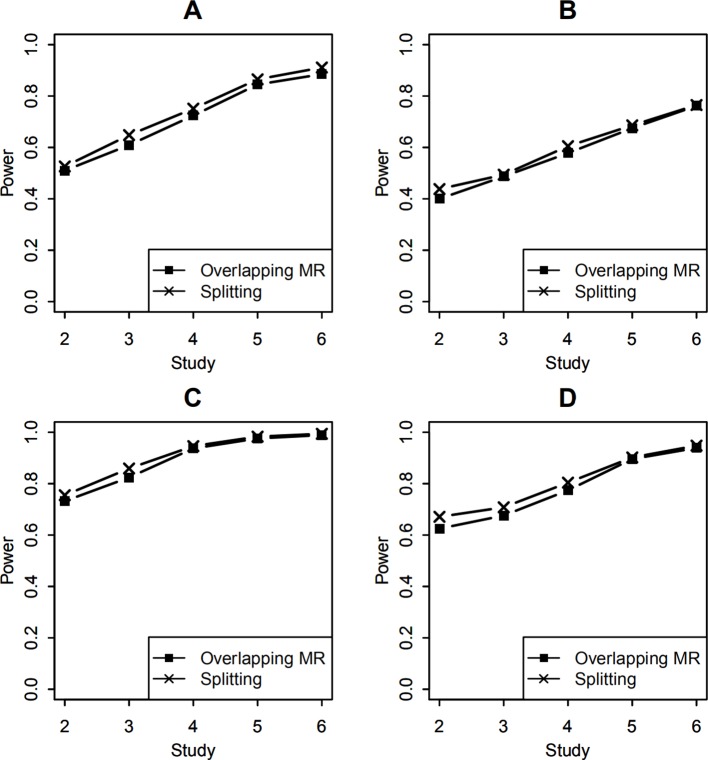
Statistical power of testing SNP-environment interaction and jointly testing SNP main effect and the interaction. **(A, B)** are statistical powers of the interaction test with 100 and 400 overlapping data, respectively. **(C, D)** are statistical powers of the joint test with 100 and 400 overlapping data, respectively. Solid line with crosses is powers of the splitting method with 2, 3, 4, 5, and 6 studies. Solid line with filled squares is powers of the OMR method with 2, 3, 4, 5, and 6 studies.

To compare the statistical power of testing the SNP-environment interaction, both SNP-environment and SNP effects explained 0.5% variance of the trait variance. In this simulation, statistical significance was determined by the P values of the tests, which were smaller than 0.05. The empirical power was obtained by calculating the proportion of the significant results in 1,000 replicates. The P values were calculated using the Wald test (9), which followed a 1 df χ^2^ distribution. **Figures 3A, B** show the power of the SNP-environment interaction with overlapping data of 100 and 400, respectively. We can see that our method yields similar results to those of the splitting method. Note that our method does not require the study-level genotype or phenotype data, which is its major advantage.

In the joint test of the SNP main effect and the SNP-environment interaction effect, both SNP-environment and SNP effects explained 0.5% variance of the trait variance. In this simulation, the P values were again calculated using the Wald test (10) following a 2 df χ^2^ distribution. **Figures 3C, D** show the powers of the joint test with 100 and 400 overlapping samples, respectively. We compared our method with the splitting method. These results are similar to those from the SNP-environment interaction test; however, the joint test yielded higher power than the interaction test. This is because the joint test included more effects than the SNP-environment interaction test (Kraft et al., 2007).

**Figure 4:**
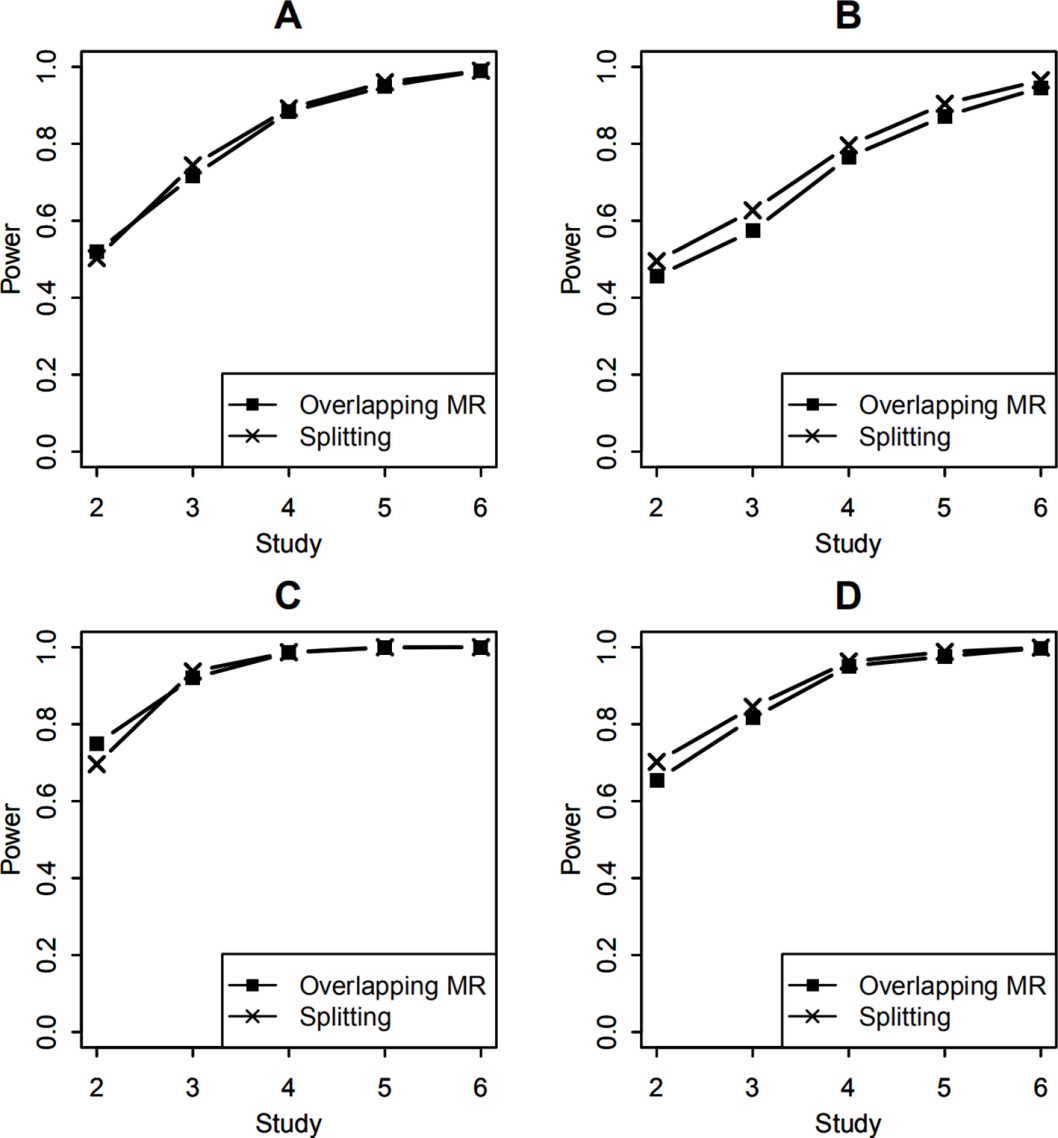
Statistical power of testing SNP-environment interaction and jointly testing SNP main effect and the interaction with 6 studies of 1,000, 1,200, 1,400, 1,600, 1,800, 2,000 individuals, respectively. **(A, B)** are statistical powers of the interaction test with 100 and 400 overlapping data, respectively. **(C, D)** are statistical powers of the joint test with 100 and 400 overlapping data, respectively. Solid line with crosses is powers of the splitting method with 2, 3, 4, 5, and 6 studies. Solid line with filled squares is powers of the OMR method with 2, 3, 4, 5, and 6 studies.

For studies with unequal sample sizes (1,000, 1,200, 1,400, 1,600, 1,800, 2,000), power of testing the SNP-environment interaction and power of the joint test for the SNP and the interaction are presented in **Figure 4**. Effects of the SNP and the interaction are the same as those in previous example. We can see that powers in **Figure 4** demonstrate similar patterns as those in **Figure 3**, whereas the former are in general larger than the latter. This is because that total sample size employed in **Figure 4** is larger than that in **Figure 3**.

**Figure 5:**
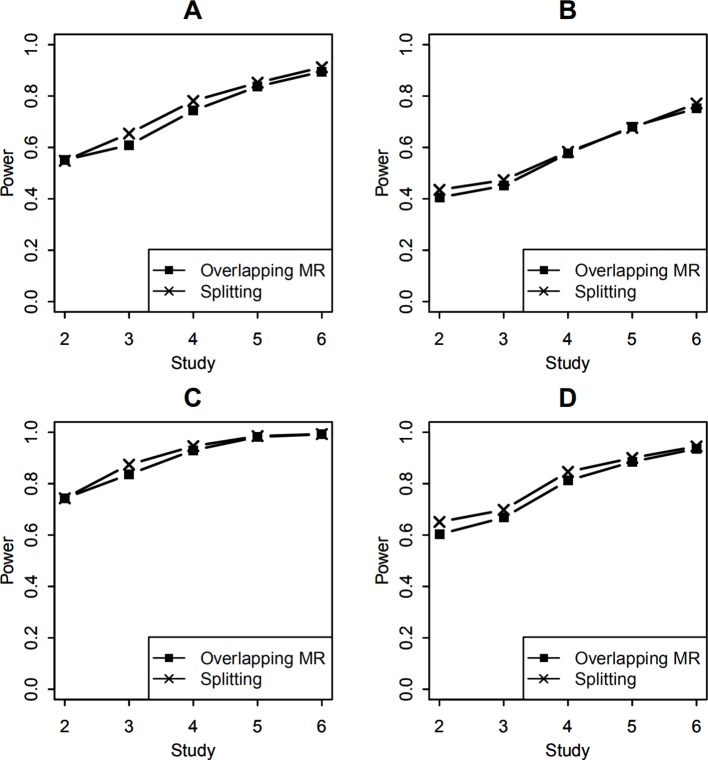
Statistical power of testing SNP-environment interaction and jointly testing SNP main effect and the interaction with opposite directions for the effects of the SNP and the SNP-environment interaction. **(A, B)** are statistical powers of the interaction test with 100 and 400 overlapping data, respectively. **(C, D)** are statistical powers of the joint test with 100 and 400 overlapping data, respectively. Solid line with crosses is powers of the splitting method with 2, 3, 4, 5, and 6 studies. Solid line with filled squares is powers of the OMR method with 2, 3, 4, 5, and 6 studies.

In GWAS, it is a common phenomenon that effects of the SNP and SNP-environment interaction may have different directions. Here, we consider the scenario that both the SNP and the interaction explained 0.5% variance of the trait variance but the directions of their effects are opposite. As in the previous example, we tested the SNP-environment interaction as well as joint effects of the SNP and the interaction. **Figures 5A, B** show powers of the interaction test with 100 and 400 overlapping samples. **Figures 5C, D** present powers of joint test with 100 and 400 overlapping samples. Compared with the results in **Figure 3**, whose effects of the SNP and interaction have the same direction, we can see that the powers of the two tests are about the same in the two scenarios.

**Figure 6:**
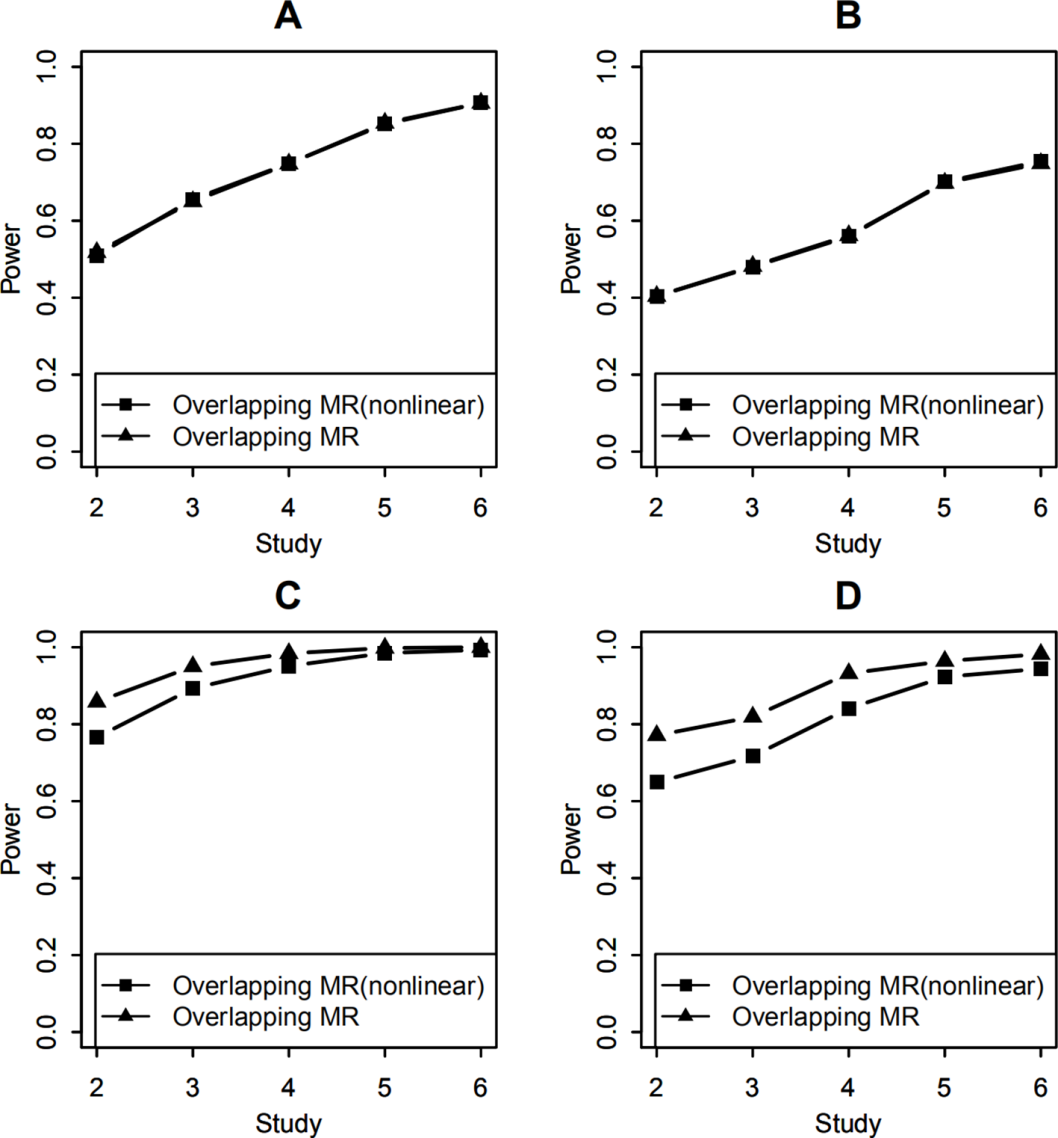
Statistical power of testing SNP-environment interaction and jointly testing SNP main effect and the interaction with nonlinear SNP-environment interaction effect. **(A, B)** are statistical powers of the interaction test with 100 and 400 overlapping data, respectively. **(C, D)** are statistical powers of the joint test with 100 and 400 overlapping data, respectively. Solid line with filled squares shows powers of OMR when nonlinear interaction effect was considered in the model. Solid line with filled triangles is powers of the OMR when nonlinear interaction effect was not considered in the model.

Finally, we added simulation for nonlinear SNP-environment interaction when testing the effect of SNP-environment interaction and the joint effects of SNP and SNP-environment. Both the effect of SNP and the effect of SNP-environment interaction explained 0.5% variance of the trait variance, the effect of nonlinear SNP-environment interaction explained 0.05% variance of the trait variance. We compared the model considering nonlinear SNP-environment as in (Xu et al., 2013). with the model not considering nonlinear SNP-environment. **Figures 6A, B** show the results of this comparison with 100 and 400 overlapping individuals for the test of interaction respectively, in each of the two figures, we can see that the two lines we compared present similar results. From **Figures 6C, D** we can see that the powers under the model considering nonlinear SNP-environment are lower than that not considering with 100 and 400 overlapping individuals for the joint test respectively. That is because the column variables in X are not an orthonormal basis when considering nonlinear interaction. The nonlinear interaction enters the model as part of the SNP main effect (Xu et al., 2013).

## Discussion

SNP may indeed interact with *E* nonlinearly in real biological process. In this case, regressing the main effect of SNP on *E* linearly involved model mis-specification. On the other hand, such linear regression can hopefully capture a portion of the main effect. In this case, we can employ Hermite polynomials to the nonlinear interaction model to avoid this phenomenon (Xu et al., 2013).

The sample sizes of studies vary in real meta-analysis. As explained in the reference (Manning et al., 2011), there are 561 individuals in the FamHS Study, 1,661 in the HealthABC Study, 2,854 in the CHS Study, 8,367 in the ARIC Study, 6,023 in the FHS Study, which gives a total sample size of 19,946. For methodological evaluations, the authors of (Manning et al., 2011) chose to simulate five studies each of 1,000 individuals. In our work, we also adopted a relatively moderate sample size 1,000 to verify the effectiveness of our method. In the revised manuscript, we conducted additional simulations to have studies with different sample sizes to evaluate the sensitivity to the unbalanced sample sizes among studies.

When testing the SNP main effect, the splitting method for case-control studies was reported to yield a lower power than Lin’s method, which is because the studies share common controls (Lin and Sullivan, 2009). Splitting these studies such that every subject contributes only once leads to a dramatic decrease in the effective sample size. Our simulation examples based on cohort studies yielded slightly less power than the splitting method because the overlapping structure in our examples differed from that in the case-control studies. The splitting method in the cohort studies drops less data than in case-control studies, so the power loss due to splitting the data is smaller.

Our method is based on the MR in which one divides the studies into several groups according to the environmental variable. Thus, when calculating the correlation matrix, we must consider both the number of overlapping data among studies and the number of overlapping data among groups. When the overlaps among groups are unavailable and the data overlap is independent of the environment variable, the overlaps between two groups can be estimated by the overlaps between their studies and the sample proportions of the groups in the two studies. In either case, our method does not require individual-level data as the splitting method does.

To the best of our knowledge, there is still no meta-analysis method for testing SNP-environment interaction with overlapping data among studies. Our OMR method was generalized from regular MR. When evaluating our proposed OMR method, we compared our method with the splitting method and regular MR. **Figure 1** indicates that regular MR yielded inflated type I error rates; the more the amount of overlapping data, the higher the amount of inflation. On the other hand, our OMR method controlled the type I error rates appropriately. Therefore, regular MR is unsuitable for studies that have overlapping data.

## Conclusion

In this paper, we generalized the regular MR model to OMR by incorporating correlations among studies due to the overlapping data. We proposed a test for the SNP-environment interaction as well as a joint test for the SNP and the interaction under the OMR framework. The two test were compared with the splitting method in terms of their type I error rate and statistical power. Through simulation, we demonstrated that our method yielded comparative power with respect to the splitting method and the type I error rate of the regular MR is inflated when overlapping data are ignored. We also evaluated our OMR method with unequal sample sizes among studies, opposite directions of the SNP effect and the interaction effect, and assessed the robustness of our method when nonlinear interaction effect exists. Our method does not require individual-level genotype and phenotype data, which overcomes the major limitation of the splitting method. In GWAS practice, our OMR method can be used to control false positive results when the studies with overlapping individuals are included in the meta-analysis, thus improve the probability of finding genuine associations.

## Data Availability Statement

The raw data supporting the conclusions of this article will be made available by the authors, without undue reservation, to any qualified researcher. 

## Author Contributions

QJ: conceived the concept, designed and conducted the simulation studies, and drafted the manuscript. GS: conceived the concept, supervised the work, and reviewed and revised the manuscript.

## Funding

This work was supported by the National Thousand Youth Talents Plan.

## Conflict of Interest

The authors declare that the research was conducted in the absence of any commercial or financial relationships that could be construed as a potential conflict of interest.
